# Complex pelvic ring injuries associated with floating knee in a poly-trauma patient: A case report

**DOI:** 10.1097/MD.0000000000008783

**Published:** 2017-12-01

**Authors:** Yuebin Zhou, Honggang Guo, Zhiwei Cai, Yuan Zhang

**Affiliations:** a Tianjin Medical University; b Department of Orthopedic Surgery, General Hospital of Tianjin Medical University, Tianjin, China.

**Keywords:** case report, complex pelvic ring injuries, damage control orthopedics, floating knee, poly-trauma

## Abstract

**Rationale:**

Complex pelvic ring fracture associated with floating knee is comparatively rare which usually results from high-energy trauma including vehicle-related accidence, falls from height, and earthquake-related injury. To our knowledge, few literatures have documented such injuries in the individual patient. Management of both injuries present challenges for surgical management and postoperative care. The purpose of this study is to prove the feasibility and benefits of damage control orthopedics (DCO).

**Patient concern:**

Our case involved a 45-year-old lady who was hit by a dilapidated building. The patient was anxious, pale and hemodynamically stable at the initial examination. The pelvis was unstable and there were obvious deformities in the left lower extremities. Significant degloved injuries in the left leg were noted. Her radiographs and physical examination verified the above signs.

**Diagnoses:**

Unstable pelvic fractures, multiple fractures of bilateral lower limbs with floating knee injury, multiple pelvic and rib fractures and multiple degloving injuries and soft tissue contusion formed the characteristics of the multiple-injury.

**Interventions:**

The algorithm of DCO was determined as the treatment. Early simplified procedures such as wound debridement, pelvis fixation, closed reduction and EF of the right shoulder joint, and chest wall fixation were conducted as soon as possible. After a period of time, internal fixations were applied to the fracture sites. The subsequent functional exercise was also conducted in accordance with this algorithm.

**Outcomes:**

This patient got recovery after the treatments which were guided by the criterion of DCO. The restoration of limb functional and the quality of life greatly improved.

**Lessons:**

The DCO plays a decisive role in the first aid and follow-up treatment of this patient. The guidelines of management of complex pelvic ring injuries and floating knee should be established by authorities.

## 1 Introduction

Pelvic ring fracture and floating knee injuries occurring in the independent damage pattern are described in medical literature,^[1,2]^ but there was no report of both injuries coexisting in the individual simultaneously. Pelvic fracture and floating knee injuries are usually caused by high-energy trauma which commonly resulted from crushing. Even with adequate intervention, the incidence of complications keeps prominent. The complications of these injuries include hemorrhage, hemodynamic instability, deep vein thrombosis, knee ligament injury, and disability of the knee joint.^[3,4]^ Therefore, appropriate therapeutic strategy should be considered in treating such injuries. Here we present a poly-trauma case with pelvic fracture and floating knee injury. Based on the injury characteristics, damage control approach was followed as the algorithm for the treatment.

## 2 Case presentation

Our case involves a 45-year-old female who was transported to our emergency center after being hit by a dilapidated building. The patient was anxious, pale, and hemodynamically stable at initial physical examination. We noted that the pelvis was rotationally unstable and there were obvious deformities and limited range of motion in the left lower extremity and right shoulder. Significant degloved injuries in the inner of left thigh and loss of 22 × 15 × 3.2 cm soft tissue in front of left tibia were noted. The Lachman and McMurray test were negative. The emergency laboratory examination indicated that the level of hemoglobin was 54 g/L, the red blood cell was 1.76 × 10^12^ L^−1^, the platelets was 54 × 10^9^ L^−1^ and the d-dimmer value was <10,000 ng/mL. Fortunately, her coagulation and renal function was normal. But the value of creatine kinase was as high as 5203 U/L which could be explained by the muscle crush injuries. The risk of death was extremely high, her ISS being as high as 29 points according to the scoring guidance.^[5]^

**Figure 1:**
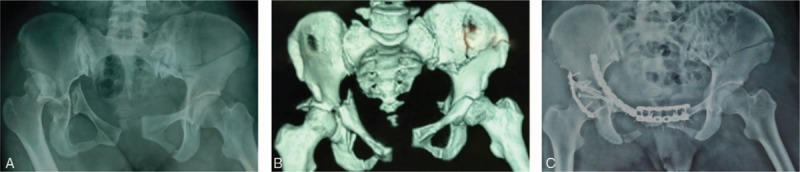
(A) This photo indicated fractures of left iliac wing, bilateral acetabulum, double ischial branch, right inferior pubic branch, and sacrum wing. The left iliac wing fracture involved the left iliac joint along with separations of right hip joint, symphysis pubis and bilateral sacral iliac joint. (B) It is the 3-dimensional CT reconstruction of broken pelvic. (C) Fractures of right acetabulum and superior ramus of pubis were fixed with porous plates and screws.

**Figure 2:**
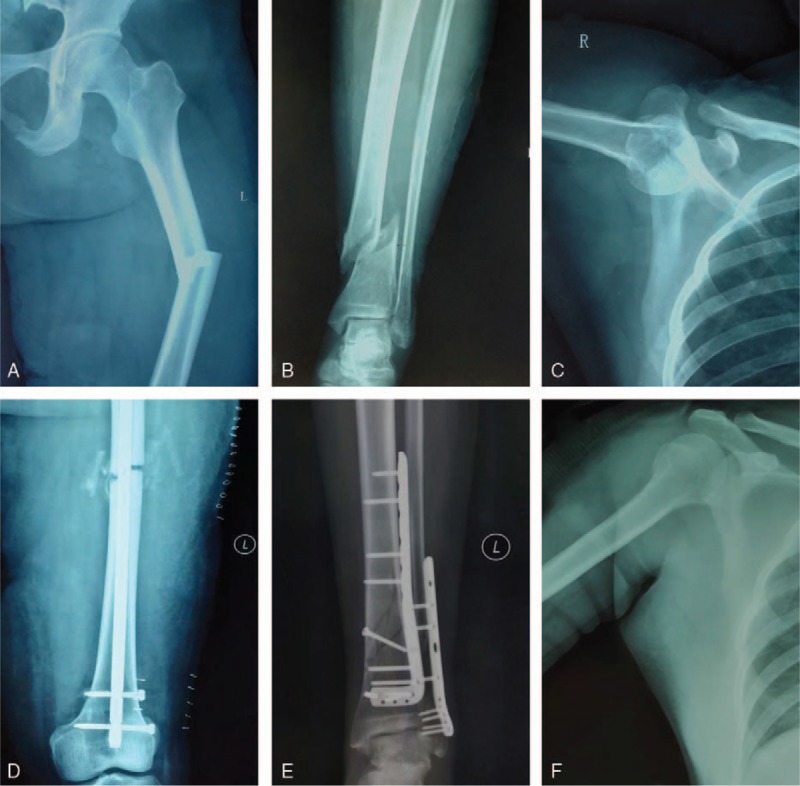
The preoperative and postoperative x-ray images of extremities and shoulder. (A) The femur was broken and two of the fractures formed an angle outwardly. (B) The tibia was fractured with the proximal fracture shifting to outside. The fibular fracture was not obvious. (C) This image shows anterior humeral head dislocation. (D) It showed the femoral shaft was fixed with intramedullary nail alone with bone grafting on broken ends and deformity corrected. (E) The tibia and fibula fractures were fixed with plates and screws, respectively. (F) The head of humerus returned to anatomical position after closed reduction.

**Figure 3:**
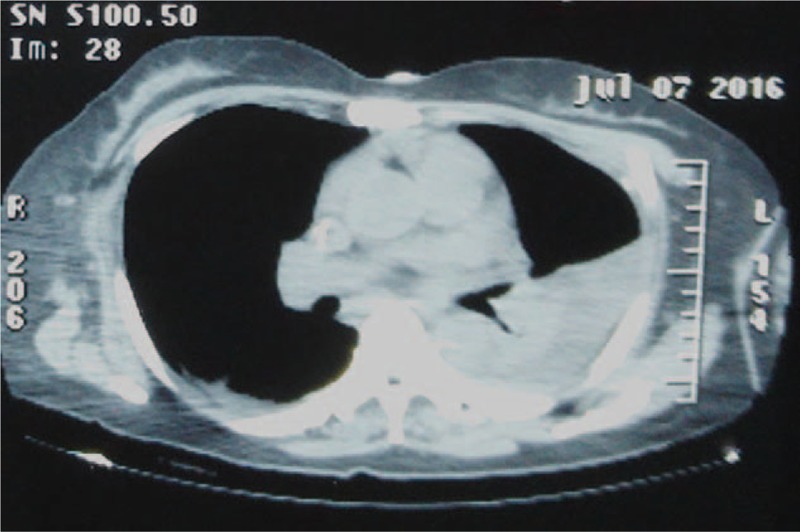
The CT scan of chest showed severe hydrothorax and atelectasis in the left lung.

Her radiographs (Fig. 1A and B) indicated fractures of left iliac wing, bilateral acetabulum, double ischial branch, right inferior pubic branch, and sacrum wing. The left iliac wing fracture involved the left iliac joint along with separations of right hip joint, symphysis pubis, and bilateral sacral iliac joint. Other pictures (Fig. 2) indicated anterior dislocation of the right shoulder, fractures of left femoral shaft, tibia and fibula and right patella. CT scan of the chest exposed severe hydrothorax in the left pleural cavity (Fig. 3). The coronal 3-dimensional CT image displayed significant instability of the pelvis with the fracture of the left iliac wing. Imaging examination ruled out abdominal organs, spinal cord, and brain trauma.

In view of the complex condition, the surgical team conducted a consultation during which the orthopedic damage control orthopedic (DCO) was determined as the algorithm for the treatment. Early simplified procedure were carried out as effective as possible to control bleeding and contamination. According to the DCO, an aseptic towel was wrapped around the pelvis to control the pelvic hemorrhage. Primary operative exploration which included wound debridement of left lower extremity, closed reduction and plaster external fixation (EF) of the right shoulder joint, and chest bands to stable the chest wall were conducted on this patient. In addition, bilateral calcaneal traction and pelvic suspension traction were imposed on her to reduce the fractures of long bones and to facilitate the reduction of the right hip separately. During the operation, 1000 mL blood plasma, 4 units of RBCs, and 2.5 L crystalloid were given to the woman to prevent coagulopathy.

After the operation, the patient was transferred to ICU for further resuscitation. Seven days later, her vital signs were stable and she was transferred back to orthopedics ward for further surgery. Unfortunately, however, Color Doppler ultrasonography showed intramuscular DVT in left extremity during the period of preoperative examination. Then inferior vena cava filter was implemented into her body by the vascular surgeons. After preoperative preparation, pelvic fracture internal fixation (Fig. 1C) was taken on the fifth day back from ICU. Three days later, open reduction and internal fixations were applied to the left femoral shaft, tibia and fibula, and right patella.

Before this case report, we have got the consent of the patient and her husband, who agreed to share their stories with others to draw attention to the prevention, first aid, and treatment of multiple-injury.

## 3 Discussions

Complex pelvic ring injuries associated with floating knee is comparatively rare; to our knowledge, there is no report concerning about it at present. Most complex pelvic ring injuries are caused by high-energy trauma, such as traffic accident or falling injury. And during the earthquake period, the earthquake-induced crush accounts for a substantial part of these cases.^[6]^

Though pelvic ring fracture is uncommon, it associates with a high risk of mortality. The mortality rate was 19% to 23%.^[6,7]^ Laszlo found that patients who had pelvic fracture-related arterial bleeding (PFRAB) had significantly higher mortality than those who had not.^[8]^ High-energy trauma such as traffic accident, falling from height, and crush injury accounts for a large part of these injuries.^[9]^ In addition, Jeff found that the presence of the pelvic ring fracture is an independent risk factor regardless of associated injuries.^[10]^ Severe hemorrhage from the pelvic ring fracture arises from several sources which include arterial injury, venous injury, and bleeding from fractured pelvis.^[11]^ At present, the primary emergency task is restoring the stability of the hemodynamics by control bleeding. Arterial injury can be controlled by therapeutic arterial embolization which Velmahos considered as an important way in the treatment of these patients.^[12]^ Bleeding from fractured bone can be controlled by EF of the pelvis.^[4]^ Also Jang found that preperitoneal pelvic packing (PPP) can be used as an effective method to control pelvic bleeding in unstable patients in their Korean trauma center.^[1]^This treatment can embolize both bleeding veins and arteries simultaneously. It may bring down the mortality associated with pelvic ring fractures effectively.

In this case, the patient had no significant bleeding when she was transferred to our center. The decline of hemoglobin in this patient may result from the fractured bone. Our surgeons gave a sterile towel to fix the pelvis temporarily when they carried out the wound debridement.

Ipsilateral fractures of the femur and tibia are called floating knee injuries which include the fractures ranging from simple diaphyseal to complex articular.^[13,14]^ Though the precise incidence of this complex injury is unknown, it is relatively uncommon.^[2,15]^ Road traffic accident is the main cause of this trauma followed by falling injury.^[16]^ With the force required to fracture 2 of the strongest bones is quiet immense, the injuries are mostly caused by high-energy trauma which may lead to extensive skeletal and soft tissue damage simultaneously. Therefore, the patients usually suffer from multiple organ injuries such as head, chest, abdomen, and limbs.^[17]^ And fewer complications and better prognosis are observed when both fractures are diaphyseal than when fractures are intra-articular.^[15]^

Floating knee injury commonly associates with ligament injuries with the incidence as high as 70.3%.^[3]^ Kumar found that ligament injury was commonly companied by either a medial or lateral meniscus injury.^[18]^ Given such hidden complications, meticulous examination of the knee and recognition of ligament and meniscus injuries are really essential.^[3]^ Surgical stabilization of both fractures, respectively, is the recommended treatment for floating knee injury currently.^[14]^ Standard practice is anterograde femoral nailing to be performed first, followed by anterograde tibia nailing.^[19]^ However, the surgical program depends on not only the fracture type but also the age, complications, patient's general conditions, and the conditions of the soft tissue and skin.

In this case, neither the articular surface nor the metaphyseal were damaged and this may benefit her functional recovery postoperatively. At first time, calcaneus traction was applied to fix the fractured bones. For the definite surgery, anterograde femoral nailing was performed first and subsequently, in view of the fracture line near to the inferior tibiofibular joint, a buttress plate was implanted to fix the tibia only. The ligaments of bilateral knee joints were not damaged in the intraoperative exploration.

Severe pelvic fractures and floating knee injury often associate with coagulation abnormality, low body temperature, and acidosis which constitute the triad of death.^[20]^ However, excessive early resuscitation may result in dilution of blood coagulation factors which may aggravate the hypothermia.^[21]^ The hypotension and traumatic shock can stimulate the body to produce multiple system inflammatory response (SIRS), which may initiate the inflammatory response and immune mechanisms.^[22]^ After the injury, the immune function strives to achieve a balance between the 2 states. If inflammatory response is too strong, it is easy to format multiple organ dysfunction syndromes (MODS); on the contrary, if anti-inflammatory reaction is too great, it is prone to lead to infection.

The damage control strategy of orthopedics (DCO) emphasizes the need to avoid further damage by the “second hit” which may originate from surgical procedures and focus on advanced life-saving supports.^[23]^ Our surgical team followed the principles of debridement and removal of the infection factors first, carried out definite surgery during the stable period. In addition to supply of the blood volume and protein, the nutritional support was emphasized.

## 4 Conclusion

Pelvic fracture associated with floating knee injury is relatively rare. However, the mortality rate is extremely high since it is often caused by high-energy injury. Appropriate principles of diagnosis and treatments play a decisive role in the treatment of these patients. In this case, the surgical team implemented segmented surgery guided by the principle of DCO. However, the treatments of this patient also have shortcomings such as lacking of dynamic monitoring of the liver and kidney function and the lactic acid levels after injury. Besides, instead of the application of EF, we just gave pelvic pocket for the pelvis and calcaneus traction for the floating knee injury, which may be unhelpful for the reduction of the fractures and bleeding control. A large sample is needed to observe the effectiveness of the diagnosis and treatment strategies.
