# Complex pelvic ring injuries associated with floating knee in a poly-trauma patient: A case report: Erratum

**DOI:** 10.1097/MD.0000000000010725

**Published:** 2018-05-11

In the article, “Complex pelvic ring injuries associated with floating knee in a poly-trauma patient: A case report”,^[1]^ which appeared in Volume 96, Issue 48 of *Medicine*, Yuebin Zhou's affiliation should have been “Department of Orthopedic, General Hospital of Tianjin Medical University, Tianjin, China.”
